# Assessment of Performance and Some Welfare Indicators of Cows in Vietnamese Smallholder Dairy Farms

**DOI:** 10.3390/ani11030674

**Published:** 2021-03-03

**Authors:** Nguyen N. Bang, Nguyen V. Chanh, Nguyen X. Trach, Duong N. Khang, Ben J. Hayes, John B. Gaughan, Russell E. Lyons, Nguyen T. Hai, David M. McNeill

**Affiliations:** 1 School of Veterinary Science, The University of Queensland, Gatton, QLD 4343, Australia; dr.russ.lyons@gmail.com; 2 Faculty of Animal Science, Vietnam National University of Agriculture, Hanoi 131000, Vietnam; nxtrach@vnua.edu.vn; 3 Faculty of Animal Science and Veterinary Medicine, Nong Lam University, Ho Chi Minh 71308, Vietnam; chanh.nguyenvan@hcmuaf.edu.vn (N.V.C.); duongnguyenkhang@gmail.com (D.N.K.); hai.nguyenthanh@hcmuaf.edu.vn (N.T.H.); 4 Queensland Alliance for Agriculture and Food Innovation, The University of Queensland, St Lucia, QLD 4067, Australia; b.hayes@uq.edu.au; 5 School of Agriculture and Food Sciences, The University of Queensland, Gatton, QLD 4343, Australia; j.gaughan@uq.edu.au

**Keywords:** smallholder dairy farms, heat stress, panting score, milk electrical resistance, artificial inseminations, dairy cow welfare

## Abstract

**Simple Summary:**

Smallholder dairy farms are the most popular type of dairy farm in Vietnam and distribute widely from north to south in both lowland and highland regions. Currently, there are limited data on productivity and especially the welfare of cows in these systems. This study was conducted on 32 farms located across four contrasting dairy regions of Vietnam to directly estimate and compare key descriptors of productivity and welfare such as herd structure, milk yield, and responses to heat stress. Level of heat stress was determined to be the most important constraint to productivity and welfare of the cows; even in the highlands where the mildest temperatures were found. Low milk yield, body weight, and body condition were also of concern. Poor productivity and welfare were most evident in the south lowland region. However, reasonable productivity in the north lowland indicated the potential to manage high temperature and humidity to improve dairy cow welfare and productivity in Vietnam.

**Abstract:**

Smallholder dairy farms (SDFs) are distributed widely across lowland and highland regions in Vietnam, but data on the productivity and welfare status of these cows remains limited. This cross-sectional study was conducted to describe and compare the productivity and welfare status of SDF cows across contrasting regions. It was conducted in autumn 2017 on 32 SDFs randomly selected from four typical but contrasting dairy regions (eight SDFs per region); a south lowland, a south highland, a north lowland, and a north highland region. Each farm was visited over a 24-h period (an afternoon followed by a morning milking and adjacent husbandry activities) to collect data of individual lactating cows (*n* = 345) and dry cows (*n* = 123), which included: milk yield and concentrations, body weight (BW), body condition score (BCS, 5-point scale, 5 = very fat), inseminations per conception, and level of heat stress experienced (panting score, 4.5-point scale, 0 = no stress). The high level of heat stress (96% of lactating cows were moderate to highly heat-stressed in the afternoon), low energy corrected milk yield (15.7 kg/cow/d), low percentage of lactating cows (37.3% herd), low BW (498 and 521 kg in lactating and dry cows, respectively), and low BCS of lactating cows (2.8) were the most important productivity and welfare concerns determined and these were most serious in the south lowland. By contrast, cows in the north lowland, a relatively hot but new dairying region, performed similarly to those in the south highland; a region historically considered to be one of the most suitable for dairy cows in Vietnam due to its cool environment. This indicates the potential to mitigate heat stress through new husbandry strategies. Cows in the north highland had the highest BW (535 and 569 kg in lactating and dry cows, respectively) and the highest energy corrected milk yield (19.2 kg/cow/d). Cows in all regions were heat-stressed during the daytime, although less so in the highlands compared to the lowlands. Opportunities for research into improving the productivity and welfare of Vietnamese SDF cows are discussed.

## 1. Introduction

Modern dairying demands the simultaneous improvement of cow productivity and welfare [1]. Productivity, as indicated mainly by milk yield and quality, drives the biological and economic efficiency of the herd. Welfare, as indicated by the physical health, mental health, and natural behaviors of the cow and provision of their basic needs such as freedom from pain, discomfort, distress, fear, hunger, and thirst, drives societal perceptions of dairying which in turn can drive market demand and price received for dairy products [1,2,3,4,5].

In Vietnam, smallholder dairy farms (SDFs) are the most popular dairy farming systems. In 2017, there were approximately 28,695 SDFs in Vietnam at last census [6], traditionally averaging 20 cows or less [7], accounting for 97% of the national dairy herd [8] and 80% of the national fresh milk production [9]. Like many tropical countries in South East Asia, the aim of dairy industry professionals and farmers is to reduce heat stress to obtain high milk yield. Consequently, SDFs in Vietnam were initially prioritized by the government to develop in the highland regions considered most suitable for taurine dairy breeds, such as Lam Dong province in the south or Son La province in the North. However, due to the increasingly high demand of fresh milk, SDFs have expanded rapidly in the relatively hot lowland provinces with major populations, such as Ho Chi Minh city in the south or Hanoi city in the north, and recently expanded to new lowland regions adjacent to the major population centers which are traditionally rice cultivating regions, such as Ha Nam province in the north or Tay Ninh in the south.

To improve the productivity and welfare of the cows it is important to record cow productivity and assess cow welfare regularly [10,11,12,13,14,15]. However, in Vietnam, SDF farmers either do not have or rarely keep herd records, such as cow pedigree, individual milk yield and composition. Additionally, there is no government regulation of dairy cow welfare in Vietnam and welfare considerations appear to have not been fully appreciated by Vietnamese farmers, dairy extension and technical advisors [16,17]. Consequently, the data on the production and welfare status of Vietnamese dairy cattle remains limited. A few reports, likely based on verbal advice from the farmer rather than direct measurement, indicate relatively low individual milk yields in the order of 14 kg/cow/d for cows in some provinces in the south [18] and 15 kg/cow/d for cows in a province in the north [19]. One of the few studies on heat stress is that of Lam et al. [20], conducted in an exceptionally hot and humid lowland region near Ho Chi Minh city at the hottest time of a year from May to June 2006. Lam et al. [20] found an average temperature-humidity index (THI) of 85 units at 1400 h, the respiration rate of 70 breaths/min (versus an unstressed target of <40 breaths/min at THI < 68, ref. [21]), and rectal temperatures of 39.3 °C (versus an unstressed target of 38.6 °C in Friesian cows ref. [22,23]). Whilst it is reasonable to expect that cow productivity and welfare are improved by locating SDFs in the cooler highland provinces such as Lam Dong in the South or Son La in the North, there has not been a study that has systematically compared the productivity and welfare of SDF cows between the highland and lowland regions. Yet the climatic records indicate that heat stress is still possible in all regions in Vietnam [24].

Realizing the limitation of available data, we conducted this study to describe and compare the current stage of productivity and welfare of SDF herds across contrasting dairy regions of Vietnam to identify opportunities for future research. Four dairy regions were selected; a south lowland (SL, Ho Chi Minh city), a south highland (SH, Lam Dong province), a north lowland (NL, Ha Nam province), and a north highland region (NH, Son La province). Anecdotal evidence from industry specialists within Vietnam is that the productivity and welfare of Vietnamese SDF cows are impaired between regions by factors including heat stress, simple diet formulations based mainly on high-fiber tropical forages such as Napier (*Pennisetum purpureum*) or rice straw plus concentrate at a ratio of 1 kg concentrate diet to 2 kg of milk yield, high cow density per farm due to lack of land, simple husbandry techniques, lack of science-based advisory support and a lack of animal welfare strategies applied [25,26,27]. We hypothesized that cows in the highland SDFs can be more productive and have better welfare than cows in the lowland SDFs in Vietnam.

## 2. Materials and Methods

### 2.1. Farm Selection

**Figure 1:**
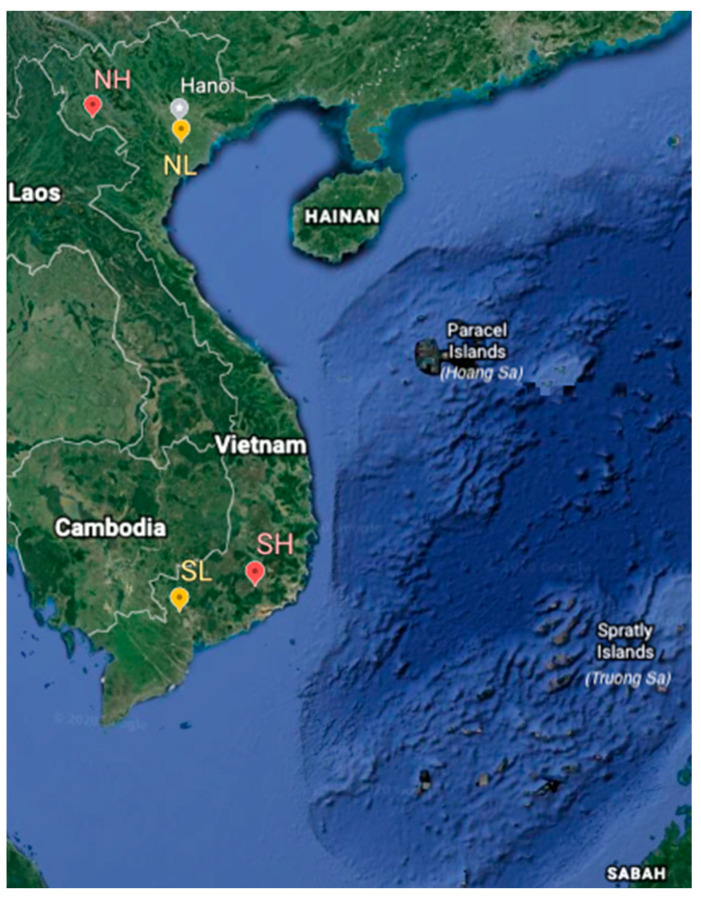
Topographic map of Vietnam and study sites including South Lowland (SL), South Highland (SH), North Lowland (NL), and North Highland (NH). Map adapted from https://www.google.com/earth/ accessed on 31 December 2019.

This cross-sectional study was conducted from 24 August to 7 October 2017 on 32 SDFs randomly selected from four main dairy regions of Vietnam, eight SDFs per region. The four main dairy regions selected included: a south lowland region (SL), which is the Cu Chi district in Ho Chi Minh city (10.82° N, 106.63° E, 5–16 m above sea level); a south highland region (SH), Don Duong district in Lam Dong province (11.58° N, 108.14° E, 800–1000 m above sea level); a north lowland region (NL), Duy Tien and Ly Nhan districts in Ha Nam province (20.58° N and 105.92° E, 0.4–12 m above sea level); a north highland region (NH), Moc Chau district in Son La province (21.33° N, 103.91° E, 600–700 m above sea level) (Figure 1). The longitude and latitude of the studied regions were derived from Google Earth Website (https://www.google.com/earth/, accessed on 31 December 2019) [28], and the altitudes were derived from the Website of Ministry of Planning and Management (http://www.mpi.gov.vn/Pages/tinhthanh.aspx, accessed on 1 January 2017) [29].

**Figure 2:**
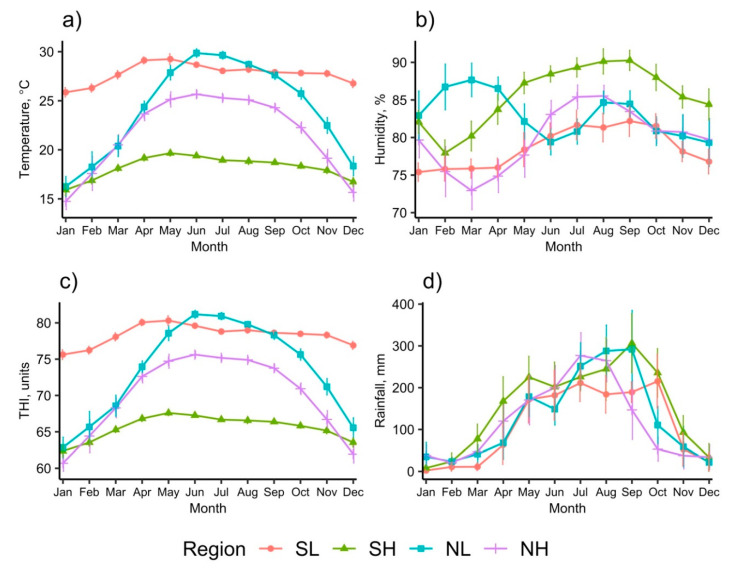
Means of daily temperature (**a**), humidity (**b**), temperature-humidity index (**c**), and rainfall (**d**) of the months of the years from 2002 to 2016. Error bars represent confident intervals.

These four regions were chosen due to a relatively long history and current popularity as a dairying area (SL, SH, NH); a short history but with recent government support for the potential to become a dairy region that embraces new technology (NL); for the contrast in the potential for heat stress provided by a high altitude (SH, NH) versus low altitude (SL, NL) environment. The data of average daily temperature, humidity, and rainfall of the months obtained from the nearest weather station during the period from 2002 to 2016, and the temperature-humidity index (THI), calculated from the temperature and humidity data using the equation of Yousef [30], are summarized in Figure 2. The current study was conducted in Autumn months which were neither the hottest nor coolest months of a year. During the study period, the temperature and THI in the SH were lowest, followed by the NH, whereas the temperature THI in the SL and SH were similarly highest.

The eight SDFs per region were randomly selected from 40 per region that had previously been included in a survey of SDF economics conducted in the same year as the current study by a collaborating scientist [31,32]. Briefly, in the economic survey, 160 SDFs with 40 per regions were randomly selected from the lists of SDFs in each region which were supplied by the local District Agriculture Departments. Then, eight SDFs per region were randomly selected from the SDFs in the economic survey which agreed to continue involving in the current study. Contact was initially made by phone to inform the nominated farmer of the purpose of the study, assure them of the voluntary and confidential nature of it, and to make an appointment for the visits which occurred on an afternoon and the following morning. The visits were designed to coincide with milking and cow feed preparation and delivery times. All farmers agreed to be involved.

### 2.2. Housing and Feeding Managements of Cows in Studied Regions

The available data on housing and feeding managements of cows in each region was limited, thus, we conducted two other studies concurrently and on the same SDFs as the current study; one on housing management [33] and other on feeding management [34]. The study on housing management showed that all surveyed SDFs were cut and carry (or zero grazing) farming systems where cows were always kept in the cowsheds [33]. All eight SDFs in the SL and five SDFs in the SH had tie-up housing where a 1–2 m length rope was threaded through a hole in cows’ nasal septum, whereas all SDFs in the NH, all SDFs in the SH, and three SDFs in the NL had loose housing; the cows could move around the pens in the cowsheds easily [33]. All SH and NH SDFs did not use any type of cooling system. NL SDFs put more effort into cooling the cows than SL SDFs by using more fans (eight fans versus one, per SDF) [33]. Additionally, two SDFs in the NL used sprinkler systems to cool the cows and seven SDFs in the NL used a roof cooling system [33]. These sprinkler and roof cooling systems were used only by SDFs in the NL but not in SDFs in any other of the regions examined [33]. Mats (mainly polyethylene foam mats) were supplied to cows similarly across SL, NL and NH SDFs (0.9 m^2^/cow), but mats were seldom used in SH SDFs.

The study on feeding management showed that the lactating cows in SL were fed mostly (on dry matter basis, DM) with commercial concentrate pellets (6.1 kg DM/cow/d), fresh Napier grass (average 2.6 kg DM/cow/d), dry rice straw (1.0 kg DM/cow/d), and fresh tropical grass (1.0 kg DM/cow/d) [34]. In SH and NL SDFs, lactating cows were fed mostly with commercial concentrate pellets (6.4 and 6.6 kg DM/cow/d for cows in SH and NL, respectively), fresh Napier grass (5.0 and 3.2 kg DM/cow/d, respectively), and corn silage (1.4 and 3.2 kg DM/cow/d, respectively) [34]. In NH SDFs, cows were fed mainly commercial concentrate pellets (7.3 kg DM/cow/d), corn silage (5.0 kg DM/cow/d), and fresh Napier grass (1.2 kg DM/cow/d) [34]. Commercial concentrate pellets fed to cows in all regions had quite similar energy and protein concentrations (1.63–1.80 MCal/kg DM and 20.4–21.7% DM of crude protein) [34]. In all regions, dry cows were fed quite similar amounts of roughages as lactating cows, but concentrate pellets were occasionally fed to dry cows [34]. Vitamins and minerals were included in commercial concentrate pellets. There was evidence that three SH SDFs, five NL SDFs, and one NH SDF used mineral supplements; one NL SDF used vitamin supplements for cows [34].

### 2.3. Collection of Productivity and Welfare Data

We visited each SDF in an afternoon and the next morning. SL SDFs were visited during the period from 24 August to 1 September; SH SDFs, periods from 5 to 9 September and 3 to 7 October; NL SDFs, period from 11 to 19 September; NH SDFs, period from 22 September to 1 October. All data were collected whilst the farmers applied their routine husbandry tasks.

At each SDF visit, the assessment team, the majority of whom visited every SDF and were trained by a visit to three practice SDFs immediately before the 32 SDFs visits, focused on the collection of data by direct observation, subsampling and measurement using a standard protocol. The individual production and welfare data of all lactating cows (*n* = 345) and dry cows (*n* = 123) across regions were collected. Due to limited labor and time, only the performance and welfare indicators that are most simple, obtainable, and applicable were selected to collect the data [2,3]. Those indicators are summarized in Table 1.

#### 2.3.1. Herd Data and Culling Reasons

The number of each livestock class—dairy bulls, lactating cows, dry cows, heifers, female calves, male calves, and beef cattle—was counted directly by the team. Per cow, age, lactation number, days in milk, and the most recent number of inseminations per conception, were obtained by checking recording books when available and asking the farmers. Estimated breed mix of both lactating and dry cows was obtained by asking farmers. While the estimated breed mix of the dry cows was obtained within the current study, the estimated breed mix of the lactating cows was obtained from another study which was conducted on the same SDFs by the same authors [44]. Common reasons to cull cows and an estimate of average herd milk yield and quality targets were also requested from the farmers. Due to insufficient recordings by farmers, we were not able to estimate culling rates in the studied herds. We were only able to ask farmers to estimate the percentages of lactating and dry cows being culled due to different reasons.

#### 2.3.2. Heart Girth and Body Weight

Heart girth (HG, kg) of each lactating cow was measured by using a commercially available tape measure [45] and draping the tape around the girth closest to the heart. Cow body weight (BW, kg) was estimated from the HG by using a recently published algorithm for cattle, BW^0.3595^ = 0.02451 + 0.04894 × HG [46].

#### 2.3.3. Body Condition Score

Body condition score (BCS) of each lactating cow was determined independently by two team members and averaged, using a 5-point system (1 for emaciated, 5 for obese with increments of 0.25) [47]. The team members were trained using the Penn State Extension video on how to score body condition (https://extension.psu.edu/learn-to-score-body-condition, accessed on 1 June 2017) [48].

#### 2.3.4. Locomotion Score

For the SDFs where the cows can walk a distance long enough to assess the locomotion score (LS), the LS of the lactating and dry cows was assessed using a scale (0–3) of DairyNZ (https://www.dairynz.co.nz/animal/cow-health/lameness/lameness-scoring/, accessed on 1 June 2017) [42]. According to DairyNZ, a cow was classified as not lame or LS = 0 when she walked evenly and confidently; slightly lame or LS = 1 when she stood with straight backline but walked unevenly with possibly arched backline; lame or LS = 2 when she stood with arched backline, walked slowly and unevenly with often arched backline, and head bobbed up and down when walking; very lame or LS = 3 when stood with arched backline, walked very slowly and very unevenly with very arched backline, and head moved up and down widely when walking [42].

#### 2.3.5. Milk Yield and Milk Electrical Resistance

As a normal practice on all SDFs, the cows were usually milked twice a day: in the early morning (04:00 h to 06:30 h) and the afternoon (15:00 h to 17:00 h), using either individual cow bucket milking machines or a vacuum pipeline system that allowed the milking of several cows simultaneously. Then all milk was bulked into 50 L milk cans and transported, often by motorbike, to the nearest milk collection center of the relevant milk processing company. Hand milking was only applied to cows with colostrum or mastitis and these cows were excluded from the lactating herd data.

For the current study, to measure a morning and next afternoon milk yield of each cow, the individual bucket was emptied between each cow milked, rather than the normal practice of letting the bucket fill from more than one cow, and for those SDFs with a vacuum pipeline system, only one cow was attached per pipeline and the milk directed to a portable 20 L container rather than the normal bulk vat. Both afternoon and next morning milk yield were weighed immediately after each cow was milked by pouring the milk into a separate bucket and weighed using a digital hanging scale that weighed to the nearest gram (Model OCS M 100, Vietnam Japan Digital Scale Company, Ho Chi Minh city, Vietnam) [49]. An approximately 40 mL subsample was then taken for further analysis (detailed later). The afternoon and next morning yields were summed to give each cow’s milk yield per day (MILK, kg/cow/d).

Milk electrical resistance (mRE, unit), as an indicator of mastitis, was measured on each 40 mL sample of milk immediately as it was taken, using a hand-held Draminski Mastitis Detector (DRAMINSKI U1. Owocowa, Olsztyn, Poland) according to the manufacturer’s instructions [41]. The mRE was calculated as the reciprocal of milk electrical conductivity. Milk samples were classified as healthy udder, mRE > 300 units; subclinically infected udder, 300 to 250 units; <250 units, clinical mastitis [41].

#### 2.3.6. Milk Sampling and Analysis

After each morning and afternoon milking of each cow, a milk sample was collected in 40 mL sterile test tubes and, immediately following the mRE test, a milk preservative tablet (18 mg tablet Broad Spectrum Microtabs II containing 8 mg Bronopol and 0.30 mg Natamycin (D & F control systems Inc., San Ramon, CA, USA) was added and mixed well into it [50]. The sample was frozen within an hour of collection and kept at −18 °C for later analysis for fat (mFA, %) by gravimetric method [51], protein (mPR, %) by Kjeldahl method [52], and dry matter (mDM, %) by standard drying method [53] at the Nutrition Laboratory, Faculty of Food Science, Vietnam National University of Agriculture. Fat yield (yFA, kg/cow/d), protein yield (yPR, kg/cow/d), and milk dry matter yield (yDM, kg/cow/d) were calculated by multiplying MILK by mFA, mPR, and mDM, respectively.

Energy-corrected milk yield (ECM, 3138 KJ per kg ECM) was calculated using the equations of Tyrrell and Reid [54]:

$$\mathrm{ECM}\;(\mathrm{kg}/\mathrm{cow}/d)\;=\frac{\mathrm{MILK}\;\left(\mathrm{kg}/\mathrm{cow}/d\right)\;\times \;\left[376\;\times \;\mathrm{mFA}\;\left(\%\right)\;+\;209\;\times \;\mathrm{mPR}\;\left(\%\right)\;+948\right]}{3138}$$

#### 2.3.7. Panting Score

Panting score (PS) of lactating cows was assessed twice a day, between 05:00 h and 06:00 h in the morning and between 1400 h and 1500 h in the afternoon, on a scale from 0 to 4.5 (0 indicates a cow breath normally, not panting; 4.5 indicates excessively panting with fast breath from the flank, tongue fully extended, excessive drooling, neck extended, and head held down) [43]). Heat stress was classified as high when PS > 1.2, moderate when 0.8–1.2, slight when 0.4–0.8, and normal when 0–0.4 [43].

### 2.4. Statistical Analyses

All statistics were performed using the base and additional packages of R software [55]. Data were imported from Microsoft Excel 2016 [56] into R using the ‘readxl’ package [57]. SDFs were the experimental units in all analyses.

Descriptive statistics for quantitative variables were calculated for each region using the ‘psych’ R package [58]. The results are presented as means for normally distributed quantitative variables, median and range for not-normally distributed quantitative variables, and frequency (percentage) for categorical variables.

The choice of suitable tests for comparisons of variables between regions was based on the guidelines of McDonald [59]. Before any statistical comparison, the normality of quantitative variables was tested using both the Shapiro–Wilk test and histogram. Variables that were found to be not-normally distributed were compared by Kruskal–Wallis tests followed by Dunn post-hoc tests (*p* < 0.05) using the ‘FSA’ R package [60]. Normally distributed variables were compared by one-way ANOVA tests followed by Tukey–Kramer tests (*p* < 0.05), using the ‘agricolae’ R package [61].

To test for associations between categorical variables, two-way contingency tables between BCS categories and regions, and between PS categories and regions were generated by using the CrossTable function of the ‘gmodels’ R package [62] and a chi-square independence test was applied. Mosaic plots generated by the ‘vcd’ R package [63] were used for visualizing the contingency tables, Pearson residuals, and independent test models which illustrate associations between categorical variables. The idea of Mosaic plots is that they recursively subdivide a unit square into rectangular “tiles” for the cells of the contingency tables, such that the area of each tile is proportional to the cell frequency [63]. The tiles are shaded in various ways to reflect the Pearson residuals (resulting from deviations of observed frequencies from expected frequencies under a given log-linear model [64]). The ‘shading_max’ function of ‘vcd’ R package [63] was used to shade the cells in the mosaic plots. The ‘shading max’ function applied the maximum statistics of the absolute Pearson residuals both to test the independence of the row and column variables in the contingency tables and to visualize significant cells which caused the rejection of the independence hypothesis [64,65]. By default, shading_max computed two cut-off points corresponding to confidence levels of 90% and 99% to shade the significant cells with less saturated colors (blue or red) and saturated colors, respectively [63]. Positive (blue) shades indicate the observed frequency is significantly greater than expected frequency under independence model which is the row variable is independent from the column variable, and negative (red) shade indicates the observed frequency is significantly less.

## 3. Results

### 3.1. Herd Characteristics

**Figure 3:**
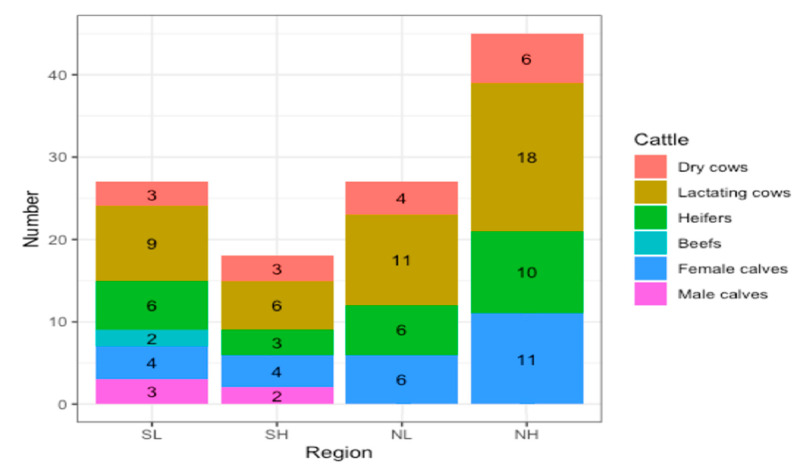
Herd structure across the four contrasting regions.

Mean herd size was largest in the NH herds (45, ranging from 34 to 55 cattle), followed by the NL (27, 16–46) and SL herds, 27 (11–44) cattle and smallest in NH herds (17, 9–30), (*p* < 0.001) (Figure 3). Across regions, herd size averaged 29 cattle, consisting of 11 lactating cows, 4 dry cows, 6 heifers, 7 female calves, 1 male calf, 1 beef cattle. There were no working bulls in any of the herds surveyed.

Herd structure was similar across regions (*p* > 0.05). The % lactating cows averaged 37.3%, dry cows 13.7%, heifers 21.4%, female calves 21.8%, male calves 3.7%, and beef cattle 1.8%.

The only region where the SDFs maintained a collective recording books of cows’ date of birth, genotype, lactation, days in milk, and reproduction data was the NH. In other regions, farmers did not have those collective recording books, they just tried to remember the information of the cows to report, thus the data on cows’ reproductivity were missing in those regions.

Table 2 represents an initial genetic and productivity categorization, LS, and reasons for culling cows, for the lactating and dry herds of each dairy region. Cow age in four regions was quite similar to each other (*p* = 0.254). Across regions, the most popular dairy breeds were 7/8 Holstein:1/8 Zebu (42%), followed by pure Holstein (31%) and 3/4 Holstein:1/4 Zebu (8%). Zebu breeds can be Red Sindhi, local Yellow (Vang) cattle, or Lai Sind (crossbreed of Red Sindhi and Yellow cattle). NH was the region where farmers reported the greatest percentage of pure Holsteins in the lactating and dry herds (100% of cows, *p* < 0.001). Whereas, for all other regions, the farmers reported that less than 19% of their lactating and dry cows were pure Holstein. SL and NL had the greatest percentages of 3/4 Holstein:1/4 Zebu and 7/8 Holstein:1/8 Zebu cows. Brown Swiss, Jersey, and 1/2 Holstein:1/2 Zebu cows were not popular in all regions.

We were only able to score the locomotion score of cows in two SL SDFs and three SH SDFs where farmers agreed to let the cow walk outside the cowsheds and in seven NH SDFs where the cows walked a distance to the milking area (Table 2). Based on the obtained data, the median percentage of lame and very lame cows (LS = 2 and 3) in NH, SH, and SL was 23%, 8%, and 7%, respectively. The median percentage of cows being culled for the range of reasons tested was similar across regions (*p* > 0.05) except for culling based on age (*p* < 0.05). The most popular reason for culling was lameness, followed by infertility, age, and mastitis. Lameness was the major reason for culling in SL cows, whilst infertility was a major reason in SH cows and NL cows. Culling on age was the major reason in NH cows.

Per lactating cow, the average number of lactations and days in milk were similar across regions (*p* > 0.05) (Table 2). Cows in all regions were artificially inseminated with semen straws of dairy breeds mainly Holstein. Whilst the average number of artificial inseminations per conception was 2.1 across regions (*p* = 0.061), herds in the SL tended to require the most (3.2), whilst herds in other regions ranged in median value from 1.6 in NH to 1.9 in NL. The mean age at first calving and mean calving interval of NH cows were 28.4 and 15.5 months, respectively.

**Figure 4:**
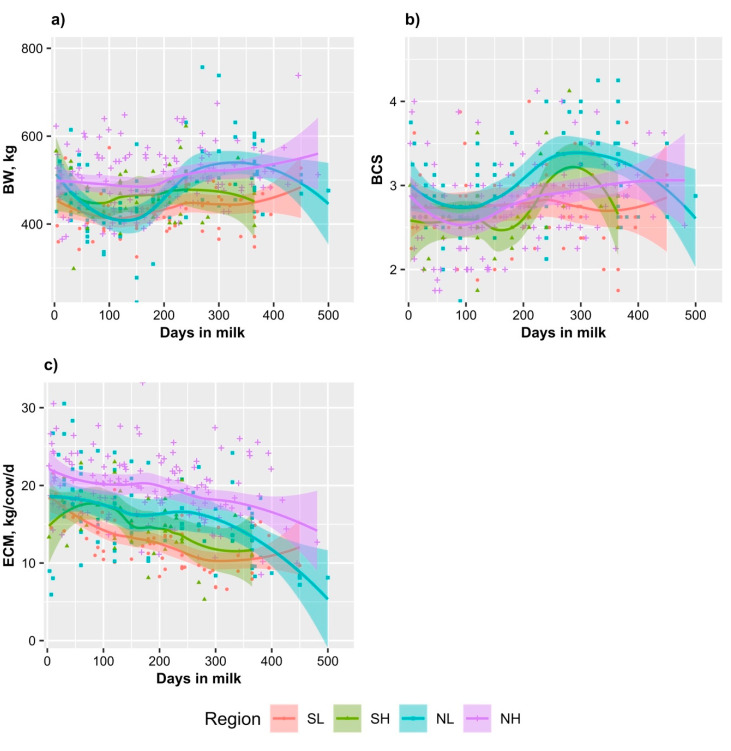
Scatter plots with smooth regression lines and 95% confidence bands of body weight (**a**), body condition score (**b**), and energy corrected milk (**c**) of lactating cows over days in milk.

Mean BW (±SEM) of cows across regions was 498 ± 18 kg for lactating cows and 521 ± 21 kg for dry cows (Table 2). BW of both lactating and dry cows were lowest in SL, highest in NH and intermediate in the NL and SH (*p* = 0.001). At the same day in milk, NH lactating cows tended to have the highest BW while SL lactating cows tended to have the lowest BW (Figure 4a).

Mean BCS (±SEM) of cows across regions was 2.8 ± 0.1 for lactating cows and 3.2 ± 0.1 for dry cows (Table 2). It was highest for NL cows, whereas all other regions were similarly lower (*p* = 0.007). Across regions, the overall percentages of lactating cows with high BCS (≥3.00), medium BCS (2.5 to 3), and low BCS (≤2.50) were 34%, 39% and 27%, respectively. Those cows with low BCS were not only the cows at early lactation when milk production was peaked, but also the cows at mid- and late lactation (Figure 4b). At a same day in milk, NL lactating cows tended to have higher BCS than that of the lactating cows in other regions (Figure 4b).

### 3.2. Milking and Milk Production

Cows in all SL, SH, and NL SDFs were milked at the cowsheds where they were normally kept during a day whilst cows in NH SDFs were often moved to a specific milking area nearby to an end of the cowshed. At milking, cows in the loose housing systems were restrained by using the headlocks whereas cows in the tie-up housing did not get any further restrain because they were already tied into the cowsheds by a rope threaded through their nasal septum. In all SDFs, cow udders were cleaned and stimulated with towels soaked in either warm or ambient-temperature water to induce milk let-down immediately before application of the milking cups. After milking, cow teats were disinfected and the milking cups of the milking machine were cleaned with clean towels. All farmers reported that calves were separated from cows right after calving and no cows were permitted to nurse calves in association with the milking process or between milking.

Mean (±SEM) MILK and ECM of cows across regions were 16.9 ± 1.5 and 15.7 ± 1.3 kg/cow/d, respectively (Table 3). The actual MILK amounts were markedly lower than the average amount targeted by farmers across regions.

Across regions, when not adjusted for BW, all milk yield parameters in NH were significantly higher than those in the southern regions (*p* ≤ 0.02, Table 3), and all milk yield parameters in SL tended to be lowest. All milk yield parameters in NL were similar to those in SH (*p* > 0.05). yPR and yPR plus yFA were higher in the NL than SL region (*p* < 0.05). At a same day in milk, ECM of NH was higher than that of cows in all other regions (Figure 4c).

When adjusted for BW, except for yFA and yDM (*p* > 0.111), milk production and quality parameters were highest for NH cows and lowest for SL cows (*p* < 0.05). The yFA and yDM were similar across regions (*p* > 0.05).

Milk component concentrations averaged across morning and afternoon and regions were: mFA, 3.66%; mPR, 3.27%; mDM, 12.32% (Table 4). Cows in highland regions tended to produce milk with lower mFA, mPR, and mDM than the cows in the lowland regions (*p* < 0.05). SL cows had the highest mFA and mDM; NL cows had the highest mPR; NH cows had the lowest mFA and mDM; SH cows had the lowest mPR (*p* < 0.05).

Milk electrical resistance (mRE), a measure of udder health, averaged across morning and afternoon and regions was 406 units. SH cows had the highest mRE and SL the lowest (*p* < 0.01) (Table 4). Only one cow, in the SL region, had an mRE less than 300 units (classified as subclinical mastitis), and one cow in the NL region which was excluded from the study due to mastitis had an mRE of less than 250 units.

All farmers across all regions were able to nominate mFA targets but only one farmer in NL and one farmer in NL were able to nominate mPR targets. Farmer-nominated mFA targets differed across regions (*p* = 0.010) (Table 4). Targets for milk solid non-fat (mDM minus mFA) were nominated by all farmers in SL, SH, and NL, but were not by farmers in NH. Across regions, mean actual daily mFA (3.66%) was consistently lower than the mean target nominated (3.8%).

### 3.3. The Heat Stress Level of Cows

For both lactating and dry cows, means of mPS and PS for cows in SL, the hottest region, were highest, whilst those for cows in SH, the coolest region, were lowest (*p* ≤ 0.004, Table 5). Mean aPS lactating cows in SH was also the lowest (*p* = 0.007). However, means of mPS, aPS, and PS of lactating and dry cows in NH were similar to those in NL, despite NH being a cooler region (*p* > 0.05).

**Figure 5:**
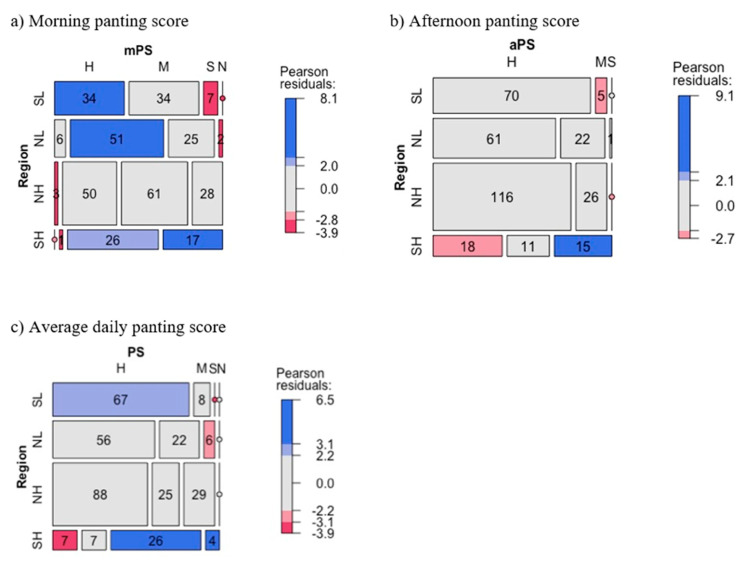
Mosaic plots showing associations between regions and panting score categories of lactating cows. (**a**) Morning panting score (mPS), (**b**) afternoon panting score (aPS), and (**c**) average daily panting score (PS). Based on panting score (PS), a cow was classified as normal (N) when PS = 0.0–0.4, slight heat stress (S) when PS = 0.4–0.8, moderate heat stress (M) when PS = 0.8–1.2, and high heat stress (H) when PS > 1.2 [43].

Figure 5 illustrates the distribution of lactating cows at different PS categories across regions. In the morning, across regions, 12% of lactating cows were highly heat-stressed, 39% were moderately, 35% were slightly, and 14% were un-stressed (Figure 5a). Only the SH region had no highly heat-stressed lactating cows. Lowland regions (NL and SL) had more (*p* < 0.01, blue rectangles) moderately (NL) to highly heat-stressed (SL) lactating cows, but less (*p* < 0.01, red rectangles) normally (both NL and SL) and slightly heat-stressed (SL) lactating cows than average.

In the afternoon, across regions, 77% of lactating cows across regions were highly heat-stressed, 19% were moderately, 4% were slightly, and none were un-stressed (Figure 5b). The SH region had a slightly lower frequency of highly heat-stressed lactating cows (41%, red rectangle, *p* < 0.10) and a higher frequency of slightly heat-stressed (34%, blue rectangle) (*p* < 0.01) than average, whereas in all other regions almost all the lactating cows were moderate to highly heat-stressed in the afternoon. The frequency of highly heat-stressed lactating cows was highest in SL with 93%, then NH with 82% and NL with 73%, and lowest in SH.

During a day, across regions, 63% of lactating cows across regions were highly heat-stressed, 18% were moderately, 18% were slightly, and 1% were un-stressed (Figure 5c). The SH region had a lower frequency of highly heat-stressed lactating cows (16%, *p* < 0.01) and higher frequencies of slightly heat-stressed (59%, *p* < 0.01) and un-stressed lactating cows (9%, *p* < 0.01) than average. The frequency of highly heat-stressed lactating cows was highest in SL with 89%, then NL with 66% and NH with 61%, and lowest in SH.

## 4. Discussion

As hypothesized, the majority of cows in both lowlands and highlands were moderately to highly heat-stressed during the day and cows in the highlands were less heat-stressed than cows in the lowlands. However, despite expectations, a similar ranking of the region was not seen for milk yield. The NH milk yields were highest but the SH milk yields were similar to the NL and SL regions. Besides heat stress, low milk yield and quality, and low BW and BCS of the SDF cows were the main productivity and welfare concerns.

### 4.1. Herd Structure and Cow Productivity

The mean herd size of Vietnamese SDFs in the current study (29 cattle) was higher than that determined in similar studies conducted in Asian and African countries. For example, in a Thai investigation, the mean herd size was 20 cows [66], in Indonesia 90% of SDFs had only three or fewer lactating cows, likewise in India 97% of SDFs had only two dairy cows [67], and in Zimbabwe the mean herd size was 10 cows [68].

Percentage of lactating cows and heifers in the whole herds were the key performance indicators of dairy farms since lactating cows generate income and replacement heifers define the sustainability of the dairy herds. Moran [69] suggested that in tropical SDFs the percentage of lactating cows should range from 40% to 48% and replacement heifers, 20–25%. Based on this guideline, the only region with an acceptable percentage of lactating cows was NL (41.1%) and possibly NH (39.1%); whereas SH (35.8%) and SL (34.8%) were lower. Mean percentage of heifers in SL (22.5%), NL (29.9%), and NH (22.3%) were all within the desirable range of 20–25% suggested by Moran [69] to ensure enough animals for replacements. Only in SH was the percentage of heifers (18.8%) lower than that suggested range.

The mean ECM of Vietnamese SDF cows (15.7 kg/cow/d) across regions in the current study is higher than the milk yield reported by previous authors based on verbal advice from the farmer rather than direct measurement, which were 14 kg/cow/d for cows in some provinces in the south [18] and 15 kg/cow/d for cows in a province in the north [19]. Mean ECM of Vietnamese SDF cows was also higher than the average yield of SDF cows in some other tropical countries such as Thai multibreed dairy cows (12.4 to 14.1 kg/cow/d) [70,71] and Indonesian Holstein cows (14.4 kg/cow/d) [72] or Ethiopian Holstein cows (11.49 kg/cow/d) [73]. However, ECM of Vietnamese SDF cows was much lower than milk yield of Holstein cows raised in the tropical commercial dairy farms of Brazil (27.59 kg/cow/d) [74] or commercial dairy farms in other developed countries such as Korea (28.6 kg/cow/d) [75], England (24.66 kg/cow/d) [76], or USA (32.50 kg/cow/d) [77]. Similarly to ECM, average yFA (0.60 kg/cow/d) of Vietnamese SDF cows was higher than yFA of Thai multibreed dairy cows (0.43–0.516 kg/cow/d) [70,71], but both yFA and yPR (0.50 kg/cow/d) of Vietnamese SDF cows lower than the ranges of yFA (0.91–1.01 kg/cow/d) and yPR (0.79–0.90 kg/cow/d) of Holstein cows in commercial farms in Brazil, Korea, and England [74,75,76].

There may be factors other than tropical weather conditions that limit the productivity of Vietnamese SDF cows. The much lower milk yields in the SH herds compared to NH herds (both with relatively cooler weather) indicated that SH SDFs might not have raised dairy cows as well as NH SDFs. Likewise, the similar milk yields in a hot region like NL compared to a region with cold weather condition like SH, and the higher yPR plus yFA in NL compared to a similar hot region like SL, suggested that the disadvantage of lowland regions in terms of hot and humid weather condition could be overcome. These results are consistent with our other studies on the same SDFs. For example, while NL SDFs put effort into cooling the cows by fans, sprinklers or roof cooling system, the SDFs in other regions especially the highland regions did not consider seriously the cooling strategies for the cows [33]. Similarly, while 88% of NH SDFs and 75% of NL SDFs supplied water ad libitum for cows, only 20% of SH SDFs and no SL SDFs supplied water ad libitum for cows [34]. These results together suggest that apart from weather conditions, other dairy farming factors such as cow genotypes, nutrition, and housing factors might have limited cow productivity. However, it should be noted that the current study was only conducted in a short period. Thus, further studies which collect data for a longer period and assess simultaneously the effects of genotypes, nutrition, and housing factors on productivity of the SDF cows are needed to clarify the effect of each of those factors. However, to assist those studies, it is important that the SDFs had to have and maintain a systematic recording books of cow diets and individual cow data including genotypes, date of birth, productivity, reproduction, and disease history, which were currently missing in all SL, NL, and SH SDFs and incomplete in NH SDFs.

The mean mFA of Vietnamese SDF cows (3.66%) was higher than that of dairy cows in some other tropical countries such as Thai multibreed dairy cows (3.19–3.59%) [70,71] or Brazilian Holstein cows (3.45%) [78], but much lower than of Holstein cows in temperate countries such as German Holstein cows (3.95–4.03%) [79] or Dutch Holstein cows (4.36%) [80]. The mean mPR of Vietnamese SDF cows (3.27%) was also higher than that of Thai multibreed dairy cows (3.01%) [81], Brazilian Holstein cows (3.05%) [78], but lower than that of German Holstein cows (3.34–3.38%) [79] and Dutch Holstein cows (3.51%) [80]. Low mFA and mPR are indicators of unbalanced diets [25]. Overall, milk component concentrations of Vietnamese SDF cows seem to be comparable with that of cows in other tropical countries, but still much lower than that of cows in temperate countries. The parameter mDM was not commonly measured by commercial dairy farms globally, but in Vietnam and other South East Asia countries such as Thailand [81], mDM along with milk solid nonfat were commonly used by some milk collecting companies to define the milk price.

### 4.2. Cow Welfare

Heat stress assessed by PS was indicated to be a serious welfare issue in Vietnamese SDF. This was especially the case in the afternoon compared to the morning, when 77% of lactating cows across all regions were categorized as suffering from high heat-stress. The high levels of heat-stressed cows in the lowlands in the current study were consistent with the study by Lam et al. [20] which reported that SDF cows in the SL were highly heat-stressed during summer with rectal temperatures of 39.3 °C (versus an unstressed target of 38.6 °C in Friesian cows; ref. [22,23]). We are not aware of any previous study assessing the level of heat stress of SDF cows in the highland regions of Vietnam. As predicted from the historical weather data [24], cows in all regions including the coolest SH region were slightly to highly heat-stressed from early morning to late afternoon. As expected, cows in highland regions suffered less heat stress than cows in lowland regions.

It is well documented that heat stress reduces feed intake and milk production in lactating cows [82,83,84], thus the high level of heat stress could be a reason for low milk productivity of Vietnamese SDF cows. This suggests that the productivity and welfare of not only cows in lowlands but also cows in highlands might be improved if their heat stress can be decreased. Interestingly, despite historical weather data between lowland regions showing that the weather conditions were quite similar during the study period, mPS and PS of cows in NL were significantly lower than that of cows in the SL. This might be because NL SDFs had better cowshed designs and applied more efficient heat stress abatement strategies than SL SDFs as mentioned previously in methodology section [33], such that the heat stress of the NL cows decreased.

Low fertility was also identified as a production issue of Vietnamese SDF cows. Medians of the number of inseminations per conception of Vietnamese SDF cows across regions, ranging from 1.6 in NH to 3.2 in SL, were considerably higher when compared to a median of 1.0 (a mean of 1.9) in Australian dairy herds reported by Talukder et al. [85] and a mean of 1.5 (range from 1.4 to 1.9) in Danish dairy herds reported by Lehmann et al. [86]. The calving interval of NH cows (15.5 months) was also considerably long. Apart from the technical factors such as heat detection, and insemination skills of inseminators, the heat stress can be a reason for high number of inseminations per conception in Vietnamese SDF cows, especially in SL regions, since it is well-documented heat stress can impair fertility and reproduction significantly in dairy cattle [87,88,89]. Avendaño-Reyes et al. [89] reported that during hot months both primiparous and multiparous cows showed significantly higher numbers of inseminations per conception and primiparous cows showed significant higher days open than in the cool months.

Average BW of Vietnamese SDF lactating cows (498 kg) and dry cows (521 kg) across four regions was much lower than BW of Holstein cows in the other countries such as Ireland [90], Brazil [91] or Israel [92]. For example, the average BW of Holstein cows raised in humid subtropical farms of Brazil ranged from 522 kg at nadir (1st parity) to 670 kg at calving (>3rd + parity) [91]. If assuming that the cow breeds reported by the farmers in the current study were true, the high percentage of crossbred cows as reported by farmers in SL, SH and NL SDFs (Table 2) is a likely reason for low average BW of Vietnamese SDF cows. This is because while Holsteins usually have high mature BW (590–680 kg), the local Vietnamese Lai Sind cattle that SDF farmers typically cross with Holsteins have very small BW (249–281 kg) [93]. Additionally, the low BW of Vietnamese SDF cows might reflect poor nutrition status of the cows. However, this hypothesis can only be confirmed if the exact breed of the cows and the common diets for Vietnamese SDF cows, which are often not recorded systematically by SDF farmers, are known.

Similar to BW, the mean BCS of lactating cows (2.8) across regions in the current study was much lower than an average BCS of 3.18 (ranging from 2.85 to 3.54) [94] or an average BCS of 3.04 (ranging from 2.88 to 3.17) [95] in US Holstein cows. It is widely accepted that BCS is a valuable indicator of cow welfare and both very lean and very fat indicates poor welfare [3,15,96]. A cow with BCS of less than 2.5 may indicate that she could be experiencing hunger [96]. In the current study, 34% of the cows across all regions had BCS less than 2.5. According to Moran and Doyle [3], a low BCS in cows, similar to low BW, is an indicator of poor welfare, and is usually due to poor feeding management. This seems to be reasonable because the available data showed that Vietnamese SDF cows are usually fed with simple diets based mainly on low-quality tropical forages such as Napier (*Pennisetum purpureum*) or rice straw, topped up with concentrate pellets at a ratio of roughly 1 kg concentrate per 2 kg of milk yield [25,26,27,97,98]. However, further research is needed to confirm this.

Lameness and infertility ranked the highest among the involuntary culling reasons and this was consistent with the results of the high number of artificial inseminations per conception and high percentage of lame and very lame cows across regions. This suggests that improving the hoof health and reproductivity of the cows are important to improve cow welfare in SDFs.

For udder health, according to the guideline of the manufacturer of the Draminski mastitis detector, cows’ udders are classified into subclinical mastitis when mRE ≤ 300 units [41]. Based on this guideline, mastitis was not a big problem in the studied regions because the means of the morning, afternoon and average day mRE over each region were all higher than 300 units. This is not consistent with a study conducted in Dong Nai, a southern province of Vietnam, which reported that the prevalence of subclinical mastitis in cows was 88.6% [99]. However, it should be noted that the diagnosis of subclinical or clinical mastitis in the current study was purely based on mRE and the guideline of the manufacturer of the mastitis detector, which is not a standard method. The accepted definition of the mastitis is that the clinical mastitis occurs when a cow produces milk with abnormal appearance and/or has swollen, red, or painful udder quarters [100]. This might explain the inconsistency in the of the prevalence of subclinical mastitis between the current study and the previous study [99].

### 4.3. Limitations

The current study had some limitations. Firstly, due to limited time and labor, only some selected performance and welfare indicators were assessed. Secondly, due to the same reasons, only single day data of productivity and welfare indicators were obtained. Thirdly, while the weather conditions of regions vary during a year and could affect productivity and heat stress level of the cows, the current study was only conducted during an autumn period. These limitations should be taken into account in further studies.

## 5. Conclusions

Comparing regions, it was as expected that cows in the highlands suffered less heat stress than cows in the lowlands. However, regarding cow productivity, only cows in the NH, but not SH, were more productive than cows in the lowlands. Future studies or extension programmes should focus more on SL SDFs where the cows yielded the least milk and were in poorest welfare conditions, and focus on SH SDFs where the weather condition was most suitable for high yielding cows but the cows were not most productive.

Across regions, the major productivity concerns of the Vietnamese SDFs were the low ECM, low percentage of lactating cows, relatively low mFA and mPR, and relatively high number of inseminations per conception. The major welfare concerns were the high heat stress level, low BW, low BCS, and culling cows due to lameness and infertility. These concerns should be targeted in further studies and extension programmes. A study which simultaneously evaluates the effects of cow genotypes, nutrition, and housing managements on those productivity and welfare indicators is necessary to point out the most potential interventions.

To improve the results, further studies are recommended to record the data for a longer period and in different seasons.

## Figures and Tables

**Table 1:** Performance and welfare indicators selected to collect the data.

Indicators	Acronym	Meaning
Percentages of cattle classes in the herd	-	Key performance indicators of a dairy farm [35,36].
Cow breed	-	Breed defines potential milk productivity and heat stress tolerability of the cows.
Cow age	-	Key performance indicators of a dairy farm [35,36].
Lactation number	-	
Days in milk	-	
Milk yield	MILK	
Milk fat concentration	mFA	
Milk protein concentration	mPR	
Milk dry matter concentration	mDM	
Energy corrected milk yield	ECM	
Culling reasons	-	A high rate of involuntary culling indicates poor welfare condition in the farms [37].
Body weight	BW	Indicators of nutritional state of the cows. Low BW or low BCS indirectly indicates undernutrition [38].
Body condition score	BCS	
Age at first calving	-	Indicators of the fertility state of the cows. High age at first calving, failure to conceive, and long calving interval are considered reduced cow production and welfare [39].
Inseminations per conception	-	
Calving interval	-	
Milk electrical resistance	mRE	An indicator of cow udder health [40,41].
Locomotion score	LS	An indicator of cow hoof health [42].
Panting score	PS	An indicator of heat stress level of cows [43].

**Table 2:** Comparisons of lactating herd characteristics, locomotion score, and reasons for culling cows between four dairy regions.

**Parameter ^A^**	**Region ^B^** **(*n* = Cows), Mean or Median**				***p*^C^**	**Overall, Mean (SEM)**
	**SL**	**SH**	**NL**	**NH**		
Lactating and dry cows	n = 98	n = 64	n = 113	n = 193		
Age, years	5.0	4.8	4.1	4.5	0.254	4.6 (0.2)
Breed categories ^D^, %						
Pure Holstein cows *	0 ^b^	19 ^ab^	6 ^b^	100 ^a^	0.001	31 (23)
7/8 Holstein:1/8 Zebu ^D,^*	63 ^a^	33 ^a^	75 ^a^	0 ^b^	0.003	42 (16)
3/4 Holstein:1/4 Zebu *	27 ^a^	6 ^ab^	0 ^b^	0 ^b^	0.002	8 (6)
1/2 Holstein:1/2 Zebu *	0	0	0	0	0.410	0 (0)
Brown Swiss and Jersey *	0	0	0	0	0.248	0 (0)
Locomotion score (LS) categories ^E^, %						
Not lame (LS = 0) *	79.00	71.00	-	53.00	0.078	68 (8)
Slight lame (LS = 1) *	14.00	22.00	-	15.00	0.780	17 (3)
Lame (LS = 2) *	7.00	8.00	-	12.00	0.179	9 (2)
Very lame (LS = 3) *	0.00	0.00	-	11.00	0.003	4 (4)
Culling reason, %						
Lameness *	45	13	0	24	0.095	21 (10)
Infertility *	20	21	17	0	0.432	15 (5)
Old *	0	8	0	31	0.046	10 (7)
Mastitis *	8	0	0	0	0.81	2 (2)
Lactating cows	n = 75	n = 44	n = 84	n = 142		
Lactation number	2.0	2.5	1.9	2.5	0.039	2.2 (0.2)
Days in milk, days	176	172	200	177	0.767	181 (7)
Heart girth, cm	179 ^b^	185 ^ab^	187 ^a^	190 ^a^	0.002	186 (2.4)
Body weight, kg	450 ^b^	496 ^ab^	513 ^a^	535 ^a^	0.001	498 (18)
Body condition score	2.7 ^b^	2.7 ^b^	3.0 ^a^	2.8 ^b^	0.007	2.8 (0.10)
Age at first calving, months	-	-	-	28.4	-	-
Calving interval, months	-	-	-	15.5	-	-
Artificial inseminations per conception *	3.2	1.7	1.9	1.6	0.061	2.1 (0.4)
Dry cows	n = 23	n = 20	n = 29	n = 51		
Heart girth, cm	186 ^b^	190 ^ab^	195 ^a^	198 ^a^	0.004	193 (3)
Body weight, kg	472 ^b^	504 ^ab^	539 ^a^	569 ^a^	0.003	521 (21)
Body condition score	3.0 ^b^	3.1 ^ab^	3.5 ^a^	3.1 ^ab^	0.043	3.2 (0.1)

^A^ Variables with (*) mark was not normally distributed, thus medians are presented; For other variables, means are presented. ^B^ Region: SL, South lowland; SH, South highland; NL, North lowland; NH, North highland. ^C^ *p* values were given for either one-way ANOVA tests comparing means (superscript letters were given for post-hoc Tukey–Kramer test, *p* < 0.05) or Kruskal–Wallis tests comparing medians (superscript letters were given for post-hoc Wilcoxon rank sum test; *p* < 0.05). ^D^ Breed data of the dry cows were obtained within the current study, but the breed data of the lactating cows were obtained from another study [44]; Zebu cattle breeds in Vietnam include Red Sindhi, Vietnamese Yellow (Vang) cattle, and Lai Sind (Red Sindhi x Yellow cattle). ^E^ Only locomotion score of cows in two SL SDFs, three SH SDFs, and seven NH SDFs were obtained and used. ^a,b^ Means or medians with the different superscript letters within a row differ significantly from each other, *p* < 0.05.

**Table 3:** Comparisons of actual milk yields (kg/cow/d), milk yields adjusted for cow body weight (kg/100 kg BW/d), and farmers’ milk yield targets (kg/cow/d) between four dairy regions.

**Parameter ^A^**	**Region ^B^ (*n* = Cows), Mean or Median**				***p*^C^**	**Overall Mean (SEM)**
	**SL (*n* = 75)**	**SH (*n* = 44)**	**NL (*n* = 84)**	**NH (*n* = 142)**		
Actual milk yields						
MILK	13.7 ^b^	16.4 ^b^	16.6 ^b^	21.0 ^a^	<0.001	16.9 (1.5)
ECM	13.1 ^b^	15.1 ^b^	15.6 ^b^	19.2 ^a^	<0.001	15.7 (1.3)
yFA	0.51 ^b^	0.56 ^b^	0.61 ^ab^	0.70 ^a^	0.002	0.60 (0.04)
yPR	0.43 ^c^	0.50 ^bc^	0.56 ^b^	0.67 ^a^	<0.001	0.50 (0.05)
yDM	1.75 ^b^	1.99 ^b^	2.05 ^ab^	2.42 ^a^	0.001	2.1 (0.14)
yFA + yPR	0.94 ^c^	1.07 ^bc^	1.18 ^ab^	1.37 ^a^	<0.001	1.1 (0.09)
Yields adjusted for BW						
ECM	2.95 ^b^	3.06 ^ab^	3.22 ^ab^	3.65 ^a^	0.024	3.3 (0.20)
yFA	0.12	0.11	0.12	0.13	0.111	0.1 (0.00)
yPR	0.10 ^b^	0.10 ^b^	0.11 ^ab^	0.13 ^a^	0.002	0.1 (0.01)
yDM	0.40	0.41	0.42	0.46	0.190	0.4 (0.01)
yFA + yPR	0.21 ^b^	0.22 ^b^	0.24 ^ab^	0.26 ^a^	0.012	0.2 (0.01)
Farmers’ milk targets						
MILK *	20.0 ^b^	25.0 ^ab^	20.5 ^ab^	29.0 ^a^	0.003	23.6 (2.1)

^A^ Abbreviations of yields per cow: MILK, milk yield; ECM, energy corrected milk yield; yFA, fat yield; yPR, protein yield; yDM, dry matter yield. Variables with (*) mark was not normally distributed, thus median are presented. For other variables, means are presented. ^B, C, a, b, c^ Other footnotes as in Table 2.

**Table 4:** Comparisons of farmers’ targets of milk concentrations (%) and measured milk concentrations (%), and milk electrical resistance between four dairy regions.

**Parameter ^A^** **, %**	**Region ^B^** **(*n* = Cows), Mean or Median**				***p*^C^**	**Overall, Mean (SEM)**
	**SL (*n* = 75)**	**SH (*n* = 44)**	**NL (*n* = 84)**	**NH (*n* = 142)**		
Actual milk concentrations						
mFA	3.92 ^a^	3.46 ^ab^	3.89 ^a^	3.38 ^b^	0.005	3.66 (0.14)
mPR	3.18 ^b^	3.10 ^b^	3.54 ^a^	3.24 ^ab^	0.004	3.27 (0.10)
mDM	12.96 ^a^	12.15 ^bc^	12.53 ^ab^	11.64 ^c^	<0.001	12.32 (0.28)
mRE	382 ^c^	431 ^a^	400 ^bc^	411 ^ab^	<0.001	406 (10)
Farmers’ targets ^D^						
mFA *	4.0	3.8	3.7	3.7	0.010	3.8 (0.1)
mPR *	3.2	-	3.3	-	-	-
mDM *	12.1	-	-	11.8	-	-
Solid non-fat *	8.7	8.8	8.7	-	0.305	8.5 (0.2)

^A^ Abbreviations: mFA, milk fat concentration (%); mPR, milk protein concentration (%); mDM, milk dry matter concentration (%). Variables with (*) mark were not normally distributed, thus medians are presented. For other variables, means are presented. ^B, C, a, b, c^ Other footnotes as in Table 2. ^D^ Only one farm in SL and one farm in NL reported milk protein target; only one farm in SL reported milk dry matter target; only one farm in NH reported milk solid non-fat target.

**Table 5:** Comparisons of the panting score of lactating and dry cows between four main dairy regions.

**Panting Score ^A^**	**Region ^B^** **(*n* = Cows), Mean**				***p*^C^**	**Overall Mean (SEM)**
	**SL**	**SH**	**NL**	**NH**		
Lactating cows	*n* = 75	*n* = 44	*n* = 84	*n* = 142		
mPS	1.4 ^a^	0.3 ^c^	0.9 ^b^	0.6 ^bc^	<0.001	0.8 (0.2)
aPS	2.2 ^a^	1.3 ^b^	1.9 ^ab^	2.0 ^a^	0.007	1.8 (0.2)
PS	1.8 ^a^	0.8 ^c^	1.4 ^b^	1.3 ^b^	<0.001	1.3 (0.2)
Dry cows	*n* = 23	*n* = 20	*n* = 29	*n* = 51		
mPS	1.3 ^a^	0.2 ^c^	1.0 ^ab^	0.6 ^bc^	<0.001	0.8 (0.2)
aPS	2.1	1.4	1.8	1.9	0.150	1.8 (0.1)
PS	1.7 ^a^	0.8 ^b^	1.4 ^ab^	1.2 ^ab^	0.004	1.3 (0.2)

^A^ Abbreviations: mPS, morning panting score; aPS, afternoon panting score; PS, average of morning and afternoon panting scores. ^B, C, a, b, c^ Other abbreviations and footnotes as in Table 2.

## Data Availability

The data presented in this study are available on request from the corresponding author.
